# Dexmedetomidine with sufentanil in intravenous patient-controlled analgesia for relief from postoperative pain, inflammation and delirium after esophageal cancer surgery

**DOI:** 10.1042/BSR20193410

**Published:** 2020-05-11

**Authors:** Chaoliang Tang, Yida Hu, Zhetao Zhang, Zeyuan Wei, Hongtao Wang, Qingtian Geng, Si Shi, Song Wang, Jiawu Wang, Xiaoqing Chai

**Affiliations:** 1 Department of Anesthesiology, The First Affiliated Hospital of USTC, Division of Life Sciences and Medicine, University of Science and Technology of China, Hefei, Anhui 230001, China; 2 Department of Anesthesiology, Renmin Hospital of Wuhan University, Wuhan, Hubei 430060, China; 3 Department of Pharmacy, The First Affiliated Hospital of USTC, Division of Life Sciences and Medicine, University of Science and Technology of China, Hefei, Anhui 230001, China

**Keywords:** delirium, Dexmedetomidine, inflammatory response, patient-controlled analgesia, postoperative pain, thoracoscopic-laparoscopic esophagectomy

## Abstract

**Background and aims:** Postoperative pain can cause serious adverse reactions that severely affect postoperative outcome. The present study evaluated the effect of dexmedetomidine (DEX) added to sufentanil in intravenous patient-controlled analgesia (PCA) on the relief of pain and inflammatory responses during postoperative recovery of patients undergoing a combined thoracoscopic-laparoscopic esophagectomy (TLE). **Methods:** Sixty patients undergoing TLE were randomly allocated to receive 1 μg/ml of sufentanil alone (Group S) or 1 μg/ml of sufentanil plus 2.5 μg/ml of DEX (Group D) for postoperative intravenous (IV) PCA. Postoperative pain relief, cumulative PCA requirements, inflammatory marker levels, delirium and recovery were assessed. **Results:** A joint DEX and sufentanil regimen significantly reduced the area under the curve of numerical rating scores for pain at rest (NRSR) and coughing (NRSC) at 1–48 h postoperatively (*P* = 0.000) that were associated with lower PCA-delivered cumulative sufentanil consumption and less PCA frequency until 48 h postoperatively (*P* < 0.05 and *P* < 0.0001, respectively). The simultaneous administration of DEX and sufentanil significantly reduced plasma IL-6 and TNF-α concentrations and increased IL-10 level (*P* < 0.0001, *P* = 0.0003 and *P* = 0.0345, respectively), accompanied by better postoperative delirium categories and health statuses of patients (*P* = 0.024 and *P* < 0.05, respectively). There was no hypotension, bradycardia, respiratory depression or oversedation in Group D. **Conclusion:** Patients receiving DEX in addition to IV PCA sufentanil for TLE exhibited better postoperative analgesia, fewer inflammatory responses and lower postoperative delirium categories and better health statuses.

## Introduction

Although combined thoracoscopic-laparoscopic esophagectomy (TLE) involves a relatively smaller incision, causes lesser pain and lesser inflammatory responses, and requires lesser recovery times compared with a traditional Ivor-Lewis esophagectomy, postoperative pain and inflammatory responses remain the common problems after esophagectomy, which severely affect patients’ postoperative recovery [1–3]. Systemic opioids in intravenous (IV) patient-controlled analgesia (PCA) are widely used; however, their unsatisfactory pain control and apparent side effects limit their application. A combination of an adjunct drug and an opioid in IV PCA is an effective regimen for pain management; it is gaining worldwide popularity in current clinical practices [4]. Dexmedetomidine (DEX) is a highly selective α_2_ receptor agonist with sedative, analgesic, anxiolytic and sympatholytic properties, and does not result in respiratory depression in clinical practice [5,6]. It has an analgesic-sparing effect, significantly reducing opioid requirements both during the intraoperative and postoperative periods [7,8], and may also decrease postoperative delirium. However, DEX is suggested as a promising option with procedure-specific, multimodal analgesia [9,10]. This may be particularly important in “enhanced recovery after surgery” protocols for patients undergoing TLE, where the reduction in pain and opioid consumption may hasten the recovery of pulmonary function, decrease the incidence of postoperative delirium and improve health status [11]. Thus, DEX in combination with other analgesic (e.g. sufentanil) during IV PCA may reduce the use of opioids and facilitate patients’ postoperative recovery.

However, the clinical use of DEX as an adjunct analgesic with sufentanil in IV PCA for relief of postoperative pain, inflammatory response and delirium during recovery of patients undergoing TLE has not been studied. We therefore conducted this prospective, randomized double-blind study in two tertiary-care hospitals in Hefei and Wuhan, China to explore the efficacy of DEX in addition to IV PCA sufentanil on relief of postoperative pain and inflammatory responses, as well as postoperative delirium categories and recovery following TLE.

## Materials and methods

### Study design

This prospective, randomized, double-blind clinical trial was approved by the Clinical Research Ethics Committees of The First Affiliated Hospital of University of Science and Technology of China and Renmin Hospital of Wuhan University of China and registered at the Chinese Clinical Trial Registry (ChiCTR, http://www.chictr.org.cn) by Chaoliang Tang (registration number, ChiCTR-TRC-14004886) on August 4, 2014. Written informed consent was obtained from all patients. Patients of either sex with American Society Anesthesiologists physical status I-III, aged between 18 and 80 years, and undergoing TLE were recruited.

### Exclusion criteria

Exclusion criteria included the following: obstructive or restrictive lung disease with FEV1/FVC% < 70%, and 50% predict ≦ FEV1 < 80% predict; asthma and sleep apnea syndrome; liver or urinary bladder disorders; known allergy to the drugs used in the study; regular use of pain perception-modifying drugs and opioids or sedative medications in the week prior to surgery; known history of second- or third-degree heart block and ischemic heart diseases; difficulties with the use of PCA; known cognitive dysfunction/dementia; and BMI >35 kg/m^2^.

### Anesthesia procedure

Patients were sent to the surgical room without any premedication 30 min before the surgery. Standard monitoring consisted of five-lead electrocardiography (ECG), oxygen saturation (SpO_2_) and non-invasive blood pressure measurements. The anesthesiologist administering the anesthetic prepared a 50-ml syringe containing 4 μg/ml of DEX. A 20-gauge intravenous cannula was inserted in the dorsum of each patient’s left hand; 0.6 μg/kg of DEX was administered, and was changed to 0.4 μg/kg/h for maintenance after 15 min. Preoxygenation with 100% oxygen was administered before induction, which was delivered through a facial mask for no less than 3 min. After the arterial line was inserted under local anesthesia, general anesthesia was induced with 0.3 mg/kg of etomidate, 0.5 μg/kg of sufentanil and 1.2 mg/kg of rocuronium. Manual facemask ventilation was continued for no less than 4 min until the jaw was relaxed and the Bispectral Index Monitoring (BIS) was less than 50 to allow the double-lumen tube intubation. As the regular thoracoscopy routine was the right in the chest, the left side of double-lumen tube was used. Auscultation and fiberoptic bronchoscopy were used to assess its correct placement. Then, the patients were connected to a mechanical ventilator with a 60% fraction of inspired O_2_ (F_i_O_2_) during a 2-lung ventilation, along with 60–100% F_i_O_2_ during 1-lung ventilation to maintain end-tidal carbon dioxide pressure (PetCO_2_) in the normal range. One percent sevoflurane was inhaled and the target-controlled anesthesia system (TCI) was used to administer remifentanil (modified Minto model, Cp 2.0–3.0 ng/ml) and propofol (modified Marsh model, Cp 2.0–3.0 μg/ml) to maintain the BIS between 40 and 60 and to ensure that the mean arterial pressure (MAP) and heart rate (HR) variation did not exceed 20% of the baseline values. Next, a central venous catheter (jugular vein), an indwelling bladder catheter and a gastric tube were inserted. Hypotension (a decrease of >20% of the baseline values) was treated with 5 mg of IV ephedrine or 40 μg of phenylephrine, while bradycardia was treated with 0.5 mg of IV atropine. The same surgical team comprising three thoracic surgeons performed all the surgeries.

A total of 1 mg/kg of tramadol and 10 mg of azasetron were administered intravenously before closing the incision, and then the administration of sevoflurane and DEX was stopped. Before the patient resumed spontaneous breathing and responded to simple commands, assistance was provided with a manual ventilator. Reversal of neuromuscular blockade was achieved with 50 μg/kg of neostigmine and 20 μg/kg of atropine. After meeting the standard extubation criteria, the patient’s double-lumen tube was removed [12].

After extubation, patients were transferred to the post anesthesia care unit (PACU) and monitored for a minimum of 1 h postoperatively. A PCA pump (ZZB-IB, Nantong AIPU Medical Inc., China) was connected to the intravenous line and configured to administer the study drug (1 ml demand dose, 10 min lockout, without background infusion). The anesthetist in the PACU, who was unaware of the clinical nature of the study, monitored the patient and prepared a 150 ml solution in the PCA reservoir bag, containing 1 μg/ml of sufentanil alone (Group S) or 1 μg/ml of sufentanil plus 2.5 μg/ml DEX (Group D). If the patient reported an NRS at rest (NRSR) of 5 or higher, the anesthetist in the PACU titrated 2 ml of the PCA solution at 5 min intervals until the Numerical Rating Scale (NRS) was 4 or less. Then, the patients were encouraged to self-administer their own PCA medications. Afterward, all patients were transferred to the intensive care unit (ICU) of the thoracic department for close monitoring over the next 48 h.

### Outcome measures

The Numerical Rating Scale (NRS) (0, no discomfort and no pain; 10, a high level of discomfort and maximum pain) [13] and the quality of recovery (QoR-15) [14], which was scored on a 11-point numerical rating scale (0–10), were explained to the patients and assessed during the preoperative visit. The QoR-15 items included: 1. “Able to breathe easy”; 2. “Been able to enjoy food”; 3. “Feeling rested”; 4. “Have had a good sleep”; 5. “Able to look after personal toilet and hygiene unaided”; 6. “Able to communicate with family or friends”; 7. “Getting support from hospital doctors and nurses”; 8. “Able to return to work or usual home activities”; 9. “Feeling comfortable and in control”; 10. “Having a feeling of general well-being”; 11. “Moderate pain”; 12. “Severe pain”; 13. “Nausea or vomiting”; 14. “Feeling worried or anxious”; 15. “Feeling sad or depressed.” Patients were randomly assigned into two study groups, Group S and Group D (*n* = 30), by random number table method, which was prepared by a statistician.

Patients were assessed at 1, 2, 4, 8, 12, 24 and 48 h after surgery. The cumulative PCA requirements and PCA frequency were recorded by the PCA machines. Pain intensity was evaluated with NRSR and NRS during coughing (NRSC). The PCA was used for at least 48 h, during which the patient’s respiratory rate, oxygen saturation and sedation score were monitored. The Modified Confusion Assessment Method (CAM)-S scores were used to diagnose postoperative delirium, which consists of four categories: normal, mild, moderate and severe [15]. Changes in the health statuses of the patients on the second day after surgery were also assessed by QoR-15. The anesthetist administering the anesthetic in the operating room, who was blinded to the group assignment, and our analgesia nurses (member of the acute pain service, APS) who did not perform anesthesia and monitoring recorded the date.

### Blood processing and analyses

On the morning of the surgery, upon arrival in the PACU, and on the second day after the surgery, 3 ml of venous blood was collected in tubes without an anticoagulant and maintained perfectly still until serum separation. The serum was precipitated by centrifugation at 4000 rpm at 4°C for 10 min, and then the supernatant was collected and placed in a −80°C cryogenic freezer for evaluation of interleukin-6 (IL-6), interleukin-10 (IL-10) and tumor necrosis factor-α (TNF-α) levels. IL-6, IL-10 and TNF-α levels were measured using Immulite automated chemiluminometer (Siemens Healthcare Diagnostics, Deerfeld, IL).

### Sample size

The power calculation for the study was based on the total postoperative use of sufentanil in the first 48 h, which was our primary outcome. A pilot study involving eight patients at our center found that the mean ± standard deviation (SD) of the total postoperative administration of sufentanil in the first 48 h was 120 ± 35 μg. In a sample size of 52 patients, a clinically significant reduction of 30% in the total postoperative use of sufentanil at a power of 90% was observed, with a two-sided significance level of 0.05. To compensate for the possibility of dropouts, we recruited a total of 60 patients, with 30 patients per group.

### Statistical analysis

The statistical analyses were performed using SPSS Statistics 22.0 software (IBM Corp., Armonk, NY, U.S.A.). All measurement indexes were checked by a normal distribution analysis. Our secondary outcome measurement was postoperative pain relief. The NRS pain scores over the first 48 postoperative hours were expressed as areas under the curve (AUC) using the trapezoid rule and were analyzed by a Mann–Whitney *U* test. The demographic characteristics data, cumulative sufentanil consumptions and PCA frequency were evaluated using an unpaired *t-*test for between-group comparisons and a paired *t*-test for within group comparisons. The χ^2^ test was used to analyze categorical variables. A Student’s *t*-test and a two-way ANOVA test were performed for unpaired quantitative variables. A *P* value < 0.05 was considered significant. For comparisons of three or more pairs, the significance level was adjusted to *P* < 0.01.

## Results

### Quantitative analysis of patients

**Figure 1:**
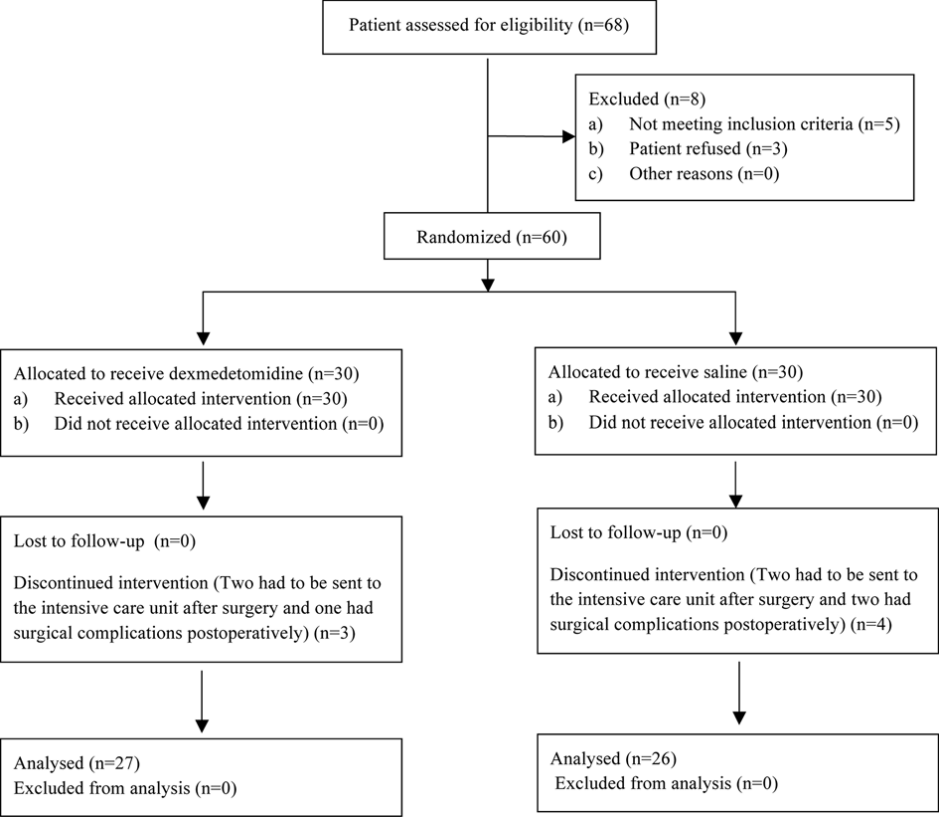
Flow diagram of patient recruitment

Sixty patients were recruited from January 2016 to August 2016 and June 2018 to April 2019. Three patients in Group D dropped out of the study, two with sustained hypoxia need breathing support had to be sent to ICU after surgery, and one had postoperative bleeding need reoperation to stop bleeding. Four patients in Group S dropped out of the study, two with sustained hypoxia need breathing support had to be sent to ICU after surgery, and two had postoperative bleeding need reoperation to stop bleeding. Fifty-three patients completed the study: 27 in Group D and 26 in Group S (Figure 1). There were no significant differences in the demographic data, surgical characteristics and intraoperative variables between the two groups (Table 1).

**Table 1:** Patient characteristics and intraoperative data

Characteristic	Treatment groups		
	Group S (*n* = 26)	Group D (*n* = 27)	*P* value
Age (year)	61.1 (8.0)	61.8 (7.5)	0.757
Male	13 (50%)	15 (56%)	0.685
Weight (kg)	66.9 (8.2)	64.7 (10.0)	0.391
Height (cm)	166.4 (9.7)	168.3 (8.9)	0.466
ASA class I/II/III	9/16/1	8/17/2	0.817
**Procedures**			
Laparoscopy (*n*)	24 (92%)	24 (89%)	0.670
Maximal laparoscopy pressure (mmHg)	13.0 (0.85)	13.2 (0.74)	0.399
Trocars (*n*)	3.0 (0.43)	3.0 (0.42)	0.942
Drainage tube (*n*)	2.2 (0.37)	2.1 (0.32)	0.654
Duration of anesthesia (min)	301.2 (45.6)	297.0 (40.0)	0.726
Duration of surgery (min)	265.8 (46.6)	268.1 (42.0)	0.851
Blood loss (ml)	168.0 (28.1)	165.3 (20.4)	0.687
Fluids (ml)	2142 (496)	2169 (595)	0.857
Urine output (ml)	546 (193)	620 (232)	0.216

Values are mean (SD) or number. All variables were similar between the two groups.

### NRSR and NRSC

**Figure 2:**
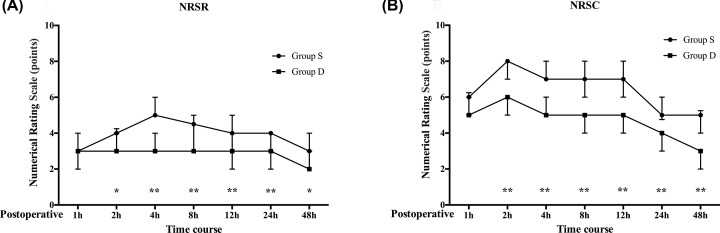
Numerical rating scores for pain at rest (NRSR) and coughing (NRSC) at 1-48 h postoperatively Postoperative numerical rating scale pain scores (**A**) at rest and (**B**) during coughing episodes during a 48 h postoperative period. Data are median with error bars showing IQR; *P<0.05, ** *P*<0.01.

NRSR and NRSC at each recorded time point are shown in Figure 2. Pain intensities were similar between groups in the first hour following surgery. However, when AUC for NRSR and NRSC pain scores for 1**–**48 h were compared, the scores were significantly lower in Group D than in Group S (*P* = 0.000) (Table 2).

**Table 2:** Postoperative area under the curve (AUC) for numerical rating scale (NRS) pain score at rest and during coughing in patients receiving dexmedetomidine or saline

Characteristic	Treatment groups		
	Group S (*n* = 26)	Group D (*n* = 27)	*P* value
Pain scores at rest			
AUC NRSR 1–60 min	3.0 (0.8)	2.7 (0.7)	0.201
AUC NRSR 1–48 h	174.3 (30.3)	122.6 (21.4)	0.000
Pain scores during coughing			
AUC NRSC 1–48 h	272.1 (42.9)	186.2 (26.3)	0.000

Values are mean (SD).

### Cumulative PCA sufentanil and PCA frequency

**Figure 3:**
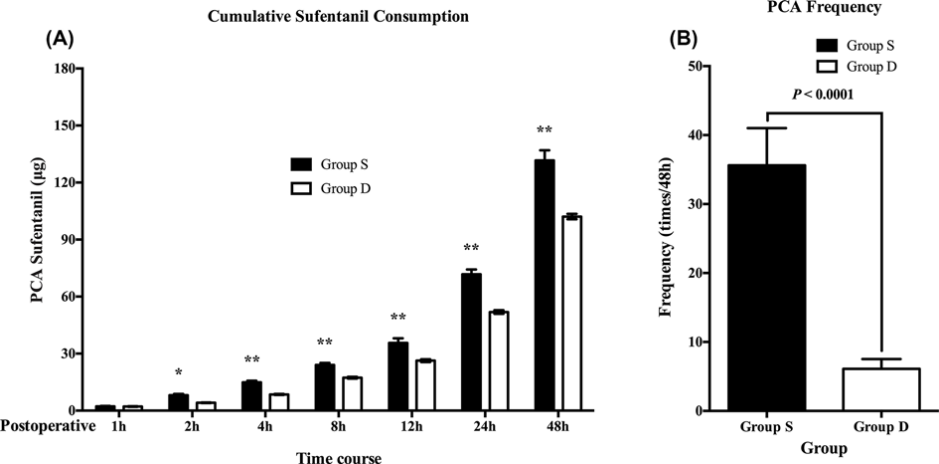
The cumulative PCA sufentanil requirements and PCA frequency Cumulative intravenous sufentanil consumption (μg) delivered by PCA (**A**) and the PCA frequency (**B**) during a 48 h postoperative period in patients receiving dexmedetomidine or saline. Values are expressed as means (95% CI); *P <0.05, **P <0.01.

Patients in Group D required significantly less PCA sufentanil than those in Group S at all times in the study. During the 0–24 h postoperative period, cumulative PCA sufentanil use was 28% more in Group S than in Group D [95% CI of difference was 16.49–23.19, *P* < 0.0001], while 22% more [95% CI of difference was 26.15–32.85, *P* < 0.0001] during the 0–48 h postoperative period. (Figure 3A). The PCA frequency was consistently significantly lesser in Group D than in Group S from the second postoperative hour onwards and throughout the study (Figure 3B). No hypotension or bradycardia was observed after PCA use.

### Postoperative delirium categories

Postoperative delirium categories based on the Confusion Assessment Method (CAM)-S scores are shown in Table 3. More patients from Group S than Group D had serious delirium. Furthermore, postoperative delirium occurred in 10 (38.5%) out of 26 patients receiving saline, and in 5 (19%) out of 27 patients receiving dexmedetomidine (*P* = 0.024).

**Table 3:** Postoperative delirium categories based on the Confusion Assessment Method (CAM)-S scores

Groups	Normal	Mild	Moderate	Severe	χ^2^	*P* value
Group S (*n* = 26)	16	1	5	4	9.399	0.024
Group D (*n* = 27)	22	4	1	0		

### Changes in the health statuses

**Figure 4:**
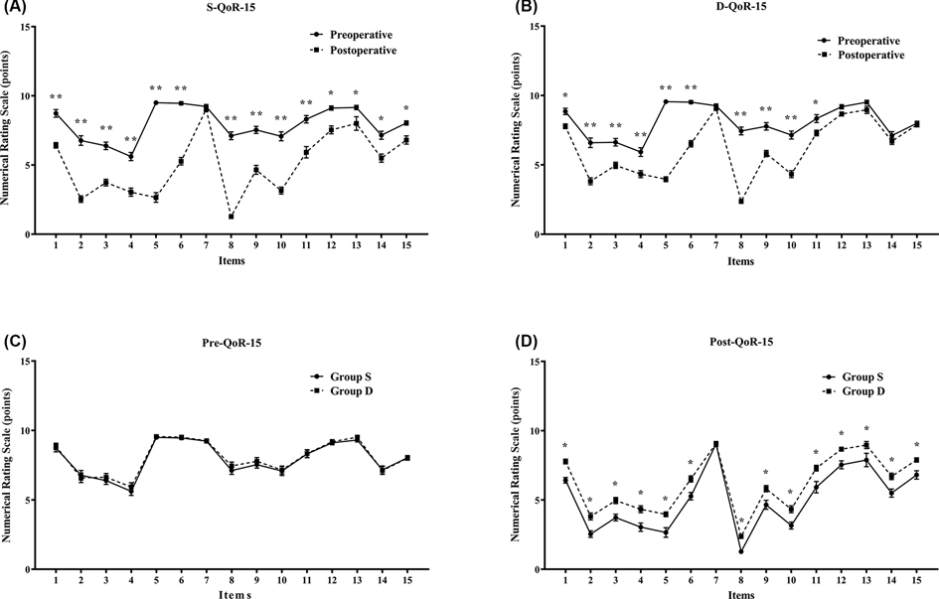
Quality of recovery by QoR-15 analysis Changes in health statuses of patients before surgery (preoperative baseline) and again on the second day after surgery (postoperative). Preoperative versus postoperative health statuses, the QoR-15 items of (**A**) Group S, (**B**) Group D; and the QoR-15 items of Group S versus Group D, (**C**) preoperative health statuses, (**D**) postoperative health statuses. Values are expressed as means (SEM); *P<0.05, **P<0.01. Quality of recovery (QoR)-15 items included the following (14): 1. ‘Able to breathe easy’; 2. ‘Able to enjoy food’; 3. ‘Feeling rested’; 4. ‘Able to sleep well’; 5. ‘Able to look after personal toilet and hygiene unaided’; 6. ‘Able to communicate with family or friends’; 7. ‘Getting support from hospital doctors and nurses’; 8. ‘Able to return to work or usual home activities’; 9. ‘Feeling comfortable and in control’; 10. ‘Having a feeling of general well-being’; 11. ‘Moderate pain’; 12. ‘Severe pain’; 13. ‘Nausea or vomiting’; 14. ‘Feeling worried or anxious’; 15. ‘Feeling sad or depressed’ (each scored on a 11-point numerical rating scale 0–10).

Changes in the health statuses of the patients before surgery (preoperative baseline) and on the second day after surgery (postoperative) are shown in Table 4. Compared with the preoperative statuses, all of the QoR-15 items of Group S, with the exception of the seventh (getting support from hospital), became significantly worse (*P* < 0.05 or *P* < 0.01), while the incidences of severe pain, nausea or vomiting and mood changes were not significantly different in Group D (Figure 4A,B). The health statuses of the patients before surgery were comparable in both groups (Figure 4C). Compared with Group S, all of the QoR-15 items, with the exception of the seventh (getting support from hospital), were significantly better in Group D (*P* < 0.05) (Figure 4D). No hypotension, or bradycardia and somnolence, or respiratory depression was reported in the present study. None of the adverse events warranted terminating PCA use.

**Table 4:** Change in health status of patients before surgery (preoperative baseline) and again on the second day after surgery (postoperative)

QoR-15 Items*	Group S (*n* = 26)		Group D (*n* = 27)	
	Preoperative	Postoperative	Preoperative	Postoperative
1. Able to breathe easy	8.73 ± 0.28	6.42 ± 0.20	8.85 ± 0.23	7.78 ± 0.17
2. Been able to enjoy food	6.77 ± 0.34	2.54 ± 0.24	6.59 ± 0.34	3.82 ± 0.25
3. Feeling rested	6.39 ± 0.28	3.73 ± 0.24	6.63 ± 0.27	4.96 ± 0.22
4. Have had a good sleep	5.61 ± 0.29	3.04 ± 0.30	5.93 ± 0.31	4.33 ± 0.25
5. Able to look after personal toilet and hygiene unaided	9.50 ± 0.10	2.65 ± 0.35	9.57 ± 0.10	3.96 ± 0.17
6. Able to communicate with family or friends	9.46 ± 0.11	5.27 ± 0.27	9.52 ± 0.11	6.52 ± 0.23
7. Getting support from hospital doctors and nurses	9.23 ± 0.14	9.04 ± 0.18	9.26 ± 0.13	9.07 ± 0.15
8. Able to return to work or usual home activities	7.12 ± 0.28	1.27 ± 0.09	7.44 ± 0.28	2.37 ± 0.14
9. Feeling comfortable and in control	7.54 ± 0.26	4.65 ± 0.32	7.78 ± 0.27	5.82 ± 0.23
10. Having a feeling of general well-being	7.08 ± 0.32	3.15 ± 0.25	7.15 ± 0.29	4.33 ± 0.25
11. Moderate pain	8.31 ± 0.27	5.92 ± 0.41	8.33 ± 0.28	7.30 ± 0.21
12. Severe pain	9.16 ± 0.15	7.54 ± 0.28	9.19 ± 0.15	8.67 ± 0.14
13. Nausea or vomiting	9.15 ± 0.15	8.00 ± 0.49	9.52 ± 0.14	8.96 ± 0.26
14. Feeling worried or anxious	7.15 ± 0.29	5.50 ± 0.30	7.11 ± 0.28	6.70 ± 0.23
15. Feeling sad or depressed	8.04 ± 0.15	6.80 ± 0.30	8.00 ± 0.14	7.89 ± 0.15
Total	120 ± 1.0	76 ± 1.3	121 ± 1.0	92.5 ± 0.9

Mean ± SEM unless otherwise stated.

* Each scored on an 11-point numerical rating scale (0–10). QoR = quality of recovery.

### IL-6, IL-10 and TNF-α plasma concentrations

**Figure 5:**
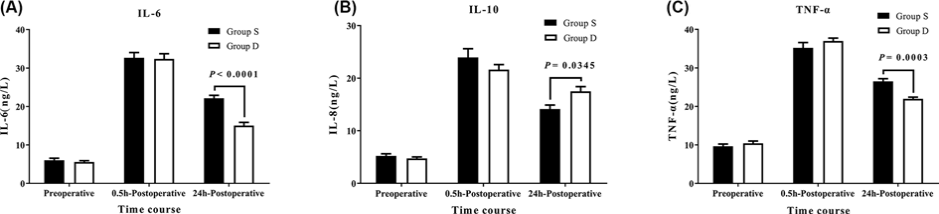
Plasma IL-6, IL-10 and TNF-α concentrations between the groups Changes in plasma (**A**) interlukin-6 (IL-6), (**B**) interlukin-10 (IL-10), and (**C**) tumor necrosis factor-α (TNF-α) levels in patients receiving dexmedetomidine or saline. Values are expressed as means ± SEM.

Plasma concentrations of IL-6, IL-10 and TNF-α were not different between the groups during preoperative and 0.5 h postoperative periods, and they all increased in both groups during the postoperative period compared with the preoperative period. IL-6 and TNF-α levels were significantly lower in Group D during the 24 h postoperative period (*P* < 0.0001 and *P* = 0.0003, respectively), while IL-10 was higher in Group D (*P* = 0.0345) (Figure 5).

## Discussion

This randomized, double-blinded comparative study was performed to evaluate the use of DEX in conjunction with sufentanil using IV PCA in TLE. Our principal findings suggest that a DEX-sufentanil mixture significantly reduces postoperative resting and pain due to coughing; enhances the analgesic effect of sufentanil; reduces the PCA sufentanil requirements and the coexisting sufentanil-induced nausea, without inducing clinically relevant bradycardia or hypotension, oversedation or respiratory depression; decreases the incidence of delirium and inflammatory responses after surgery; and improves patient recovery.

The opioid-sparing effects of DEX have been studied in conscious healthy volunteers and surgical patients [8,13,16,17]. Our finding that patients receiving DEX required 28% less sufentanil via PCA, provided additional evidence for the anesthetic-sparing effect of DEX in clinical practice, which was also associated with reduced postoperative pain in our present study. However, previous studies demonstrated that intravenous DEX has a postoperative opioid-sparing effect but has no impact on the reduction of pain [18]. In the present study, all patients were encouraged to push the PCA button to achieve an equal NRSR ≤ 4 between 2 and 48 h after the surgery. Patients receiving a DEX-sufentanil mixture administered via PCA experienced significantly lower AUCs for NRSR and NRSC pain scores during a 1–48 h postoperative period. In the present study, DEX also significantly improved the subjective quality of sleep, while sedation levels were similar between the groups. An analgesic action, rather than a sedative effect, was more likely to correspond for the reduced sufentanil requirements by DEX. The different effects of DEX on postoperative pain between our study and previous studies may be due to different combinations of other anesthetics with DEX, and may also be due to the different doses of DEX used in those studies [19,20].

The main concern related to DEX administered via IV PCA is that it is unnecessary or that it may lead to excessive sedation. Fortunately, there was no evidence of an excessive sedative effect of DEX during the course of PCA used in the present study. This may be because the doses of DEX used in conjunction with sufentanil via PCA were well within a quarter range of the recommended maintenance infusion (0.2–0.7 μg/kg/h) for intensive care sedation [21–23]. These sedative doses of DEX were associated with adverse events, especially hypotension and bradycardia [24]. In addition, the reduced cumulative sufentanil requirements administered via PCA may also play an important role in mitigating sedation. In contrast, the PCA-based drug delivery system, which has its own safe individual drug titration, may also help minimize sedation [25].

The pro-inflammatory cytokines (PICs), such as IL-6 and TNF-α, and the anti-inflammatory cytokine, IL-10, are important groups of inflammatory mediators and play an essential role in pain sensitization [26–28]. Systemic or regional analgesic regimens could prevent both peripheral and central sensitization, thereby attenuating the postoperative amplification of pain sensation [16,29,30]. Significantly increased IL-6, IL-10 and TNF-α serum levels were detected in our patients during the 0.5 h postoperative period in both groups. Group D had lower pro-inflammatory cytokine levels and higher anti-inflammatory cytokine levels compared with Group S during the 24 h postoperative period. This result was consistent with our findings of postoperative NRSR and NRSC pain scores, which indicated that Group D had significantly lower NRSR and NRSC pain scores during the 2–48 h postoperative period than Group S. Our results suggest that a DEX–sufentanil mixture administered via PCA would be effective in reducing an inflammatory response, which would also reduce postoperative pain sensitization.

Postoperative delirium, which is characterized by dysfunction in consciousness, attention and cognition, is always regarded as a reversible cognitive dysfunction syndrome [31]. It is a common complication in elderly patients, especially 1–3 days after a surgery. Excessive and prolonged postoperative delirium may influence a patient’s recovery [32–34]. In the present study, we used CAM-S scores, a quite rigorous assessment system, to diagnose postoperative delirium. Furthermore, postoperative delirium occurred in 6 (23%) out of 26 patients receiving saline, similar to previous studies, [35–37] and in 5 (19%) out of 27 patients receiving DEX. Although the incidence of postoperative delirium may not be significant, patients receiving DEX had lower moderate and severe categories of postoperative delirium compared with patients receiving saline (1 and 0 vs 5 and 4). A higher PCA frequency resulted in patients receiving saline also receiving supplemental sufentanil, which might increase the risk of postoperative delirium [38]. The physiopathologic mechanism of how DEX decreases delirium remains unknown. However, its opioid-sparing and anti-inflammatory effects may provide good indications [39,40]. A good number of reasons may explain how DEX decreased postoperative delirium in our study. First, DEX intervention was initiated as soon as patients were transferred to the PACU, which prevented delirium during the early postoperative hours [41]. Second, we used DEX during the 48 h postoperative period, which improved patients’ sleep quality in the evening, since a central effect of DEX converges with an endogenous sleep-promoting pathway [42].

A DEX-sufentanil mixture administered via PCA also had a beneficial effect on the recovery of bowel function and ambulation, which may have been associated with lower sufentanil consumption. Large doses of sufentanil may inhibit intestinal motility. A jejunum colostomy indicated that the incidence of nausea or vomiting was directly associated with the discomfort caused by a gastric tube and duodenal feeding tube in both groups. However, patients receiving DEX still showed less nausea or vomiting and fewer side effects from opioids. In addition, the pain caused by chest tubes and ribcage expansion may adversely affect coughing and deep breathing, which may result in respiratory complications and delay recovery [2]. On the contrary, reliable analgesia may facilitate deep breathing, clearance of secretions, promote active participation in physiotherapy and reduce respiratory complications.

There are several limitations in the present study. Although the study had a modest sample size that achieved significant differences in endpoints between the two groups, this sample size was not specifically powered to detect the effects of DEX in postoperative delirium. Furthermore, it has been noted that CAM-S might not be as sensitive as other tools, e.g. 3D-CAM, for delirium assessment, especially for other ethnic groups [43–45]. Although it is stated that in Enhanced recovery programmers (ERP) minimization of opioid administration is key, recently, paravertebral block (PVB) is also recognized as vital to reduce amount of opioids and minimize sedation and constipation in thoracic ERP [2]. And thoracic PVB with the addition of dexmedetomidine could exhibit more improved quality and duration of analgesia, as well as an analgesic sparing effect with no serious side effects [8]. Thus, future studies with larger sample sizes; more sensitive delirium assessment tools and groups with local anesthesia techniques are needed.

## Conclusion

In summary, our study indicates that a DEX–sufentanil mixture administered via PCA after esophageal cancer surgery resulted in a reduction in pain intensities at rest and during coughing episodes. In addition, a decrease in PCA sufentanil requirements resulted in reduced nausea induced by sufentanil. Using a PCA-based drug administration, DEX appears to be a promising and safe adjunct in postoperative pain control in esophageal cancer surgery, since it eliminates unwanted oversedation, attenuates inflammatory responses, implicates positive effects on postoperative delirium, and improves the quality of analgesia and recovery.

## Data Availability

The data used to support the findings of this study are available from the corresponding author upon request.

## Abbreviations

APS: acute pain service
AUC: area under the curve
BIS: bispectral index monitoring
CAM-S: modified confusion assessment method scores
DEX: dexmedetomidine
ECG: electrocardiography
ERP: enhanced recovery programmers
FiO_2_: fraction of inspired O_2_
HR: heart rate
ICU: intensive care unit
IL: interleukin
IV: intravenous
MAP: mean arterial pressure
NRS: numerical rating scale
NRSC: numerical rating scores for pain at coughing
NRSR: numerical rating scores for pain at rest
PACU: post anesthesia care unit
PCA: patient-controlled analgesia
PetCO_2_: end-tidal carbon dioxide pressure
PICs: pro-inflammatory cytokines
PVB: paravertebral block
QoR: quality of recovery
SD: standard deviation
SpO_2_: oxygen saturation
TCI: target-controlled anesthesia system
TLE: thoracoscopic-laparoscopic esophagectomy
TNF-α: tumor necrosis factor-alpha

## References

[1] Kinjo Y., Kurita N., Nakamura F., Okabe H., Tanaka E., Kataoka Y. et al. (2012) Effectiveness of combined thoracoscopic-laparoscopic esophagectomy: comparison of postoperative complications and midterm oncological outcomes in patients with esophageal cancer. Surg. Endosc 26, 381–90 10.1007/s00464-011-1883-y 21898014

[2] Zhang W., Fang C., Li J., Geng Q.T., Wang S., Kang F. et al. (2014) Single-dose, bilateral paravertebral block plus intravenous sufentanil analgesia in patients with esophageal cancer undergoing combined thoracoscopic-laparoscopic esophagectomy: a safe and effective alternative. J. Cardiothorac. Vasc. Anesth. 28, 966–72 10.1053/j.jvca.2013.12.007 24686029

[3] Wei K., Min S., Hao Y., Ran W. and Lv F. (2019) Postoperative analgesia after combined thoracoscopic-laparoscopic esophagectomy: a randomized comparison of continuous infusion and intermittent bolus thoracic epidural regimens. J. Pain Res. 12, 29–37 10.2147/JPR.S188568 30588077 PMC6302820

[4] Song J.W., Shim J.K., Song Y., Yang S.Y., Park S.J. and Kwak Y.L. (2013) Effect of ketamine as an adjunct to intravenous patient-controlled analgesia, in patients at high risk of postoperative nausea and vomiting undergoing lumbar spinal surgery. Br. J. Anaesth. 111, 630–5 10.1093/bja/aet192 23744819

[5] Hsu Y.W., Cortinez L.I., Robertson K.M., Keifer J.C., Sum-Ping S.T., Moretti E.W. et al. (2004) Dexmedetomidine pharmacodynamics: part I: crossover comparison of the respiratory effects of dexmedetomidine and remifentanil in healthy volunteers. Anesthesiology 101, 1066–76 10.1097/00000542-200411000-00005 15505441

[6] Cortinez L.I., Hsu Y.W., Sum-Ping S.T., Young C., Keifer J.C., Macleod D. et al. (2004) Dexmedetomidine pharmacodynamics: Part II: Crossover comparison of the analgesic effect of dexmedetomidine and remifentanil in healthy volunteers. Anesthesiology 101, 1077–83 10.1097/00000542-200411000-00006 15505442

[7] Ramsay M.A. and Luterman D.L. (2004) Dexmedetomidine as a total intravenous anesthetic agent. Anesthesiology 101, 787–90 10.1097/00000542-200409000-00028 15329604

[8] Tang C. and Xia Z. (2017) Dexmedetomidine in perioperative acute pain management: a non-opioid adjuvant analgesic. J. Pain Res. 10, 1899–904 10.2147/JPR.S139387 28860845 PMC5565238

[9] Lui F. and Ng K.F. (2011) Adjuvant analgesics in acute pain. Exp. Opin. Pharmacother. 12, 363–85 10.1517/14656566.2011.521743 21254945

[10] Nicacio I.P., Stelle A.B.F., Bruno T.S., Nicacio G.M., Costa J.S. Jr and Cassu R.N. (2020) Comparison of intraperitoneal ropivacaine and ropivacaine-dexmedetomidine for postoperative analgesia in cats undergoing ovariohysterectomy. Vet. Anaesth. Analg. 47, 396–404 10.1016/j.vaa.2020.01.007 32199795

[11] Morris C. and Gold S. (2011) Enhanced recovery programmes; coming to a hospital near you!. Anaesthesia 66, 864–8 10.1111/j.1365-2044.2011.06883.x 21916861

[12] Tang C., Li J., Lei S., Zhao B., Zhang Z., Huang W. et al. (2017) Lung-Protective Ventilation Strategies for Relief from Ventilator-Associated Lung Injury in Patients Undergoing Craniotomy: A Bicenter Randomized, Parallel, and Controlled Trial. Oxid Med. Cell. Longev. 2017, 6501248 10.1155/2017/6501248 28757912 PMC5516714

[13] Cheung C.W., Qiu Q., Liu J., Chu K.M. and Irwin M.G. (2015) Intranasal dexmedetomidine in combination with patient-controlled sedation during upper gastrointestinal endoscopy: a randomised trial. Acta Anaesthesiol. Scand. 59, 215–23 10.1111/aas.12445 25471688

[14] Stark P.A., Myles P.S. and Burke J.A. (2013) Development and psychometric evaluation of a postoperative quality of recovery score: the QoR-15. Anesthesiology 118, 1332–40 10.1097/ALN.0b013e318289b84b 23411725

[15] Inouye S.K., Kosar C.M., Tommet D., Schmitt E.M., Puelle M.R., Saczynski J.S. et al. (2014) The CAM-S: development and validation of a new scoring system for delirium severity in 2 cohorts. Ann. Intern. Med. 160, 526–33 10.7326/M13-1927 24733193 PMC4038434

[16] Tang C., Huang X., Kang F., Chai X., Wang S., Yin G. et al. (2015) Intranasal Dexmedetomidine on Stress Hormones, Inflammatory Markers, and Postoperative Analgesia after Functional Endoscopic Sinus Surgery. Mediators Inflamm. 2015, 1–9 10.1155/2015/939431 PMC4496499 26199465

[17] Lin T.F., Yeh Y.C., Lin F.S., Wang Y.P., Lin C.J., Sun W.Z. et al. (2009) Effect of combining dexmedetomidine and morphine for intravenous patient-controlled analgesia. Br. J. Anaesth. 102, 117–22 10.1093/bja/aen320 18987053

[18] Chan A.K., Cheung C.W. and Chong Y.K. (2010) Alpha-2 agonists in acute pain management. Exp. Opin. Pharmacother. 11, 2849–68 10.1517/14656566.2010.511613 20707597

[19] Nie Y., Liu Y., Luo Q. and Huang S. (2014) Effect of dexmedetomidine combined with sufentanil for post-caesarean section intravenous analgesia: a randomised, placebo-controlled study. Eur. J. Anaesthesiol. 31, 197–203 10.1097/EJA.0000000000000011 24463478

[20] Wang X., Wang K., Wang B., Jiang T., Xu Z., Wang F. et al. (2016) Effect of Oxycodone Combined With Dexmedetomidine for Intravenous Patient-Controlled Analgesia After Video-Assisted Thoracoscopic Lobectomy. J. Cardiothorac. Vasc. Anesth. 30, 1015–21 10.1053/j.jvca.2016.03.127 27521970

[21] Pandharipande P.P., Pun B.T., Herr D.L., Maze M., Girard T.D., Miller R.R. et al. (2007) Effect of sedation with dexmedetomidine vs lorazepam on acute brain dysfunction in mechanically ventilated patients: the MENDS randomized controlled trial. JAMA 298, 2644–53 10.1001/jama.298.22.2644 18073360

[22] Xia Z.Q., Chen S.Q., Yao X., Xie C.B., Wen S.H. and Liu K.X. (2013) Clinical benefits of dexmedetomidine versus propofol in adult intensive care unit patients: a meta-analysis of randomized clinical trials. J. Surg. Res. 185, 833–43 10.1016/j.jss.2013.06.062 23910886

[23] Riker R.R., Shehabi Y., Bokesch P.M., Ceraso D., Wisemandle W., Koura F. et al. (2009) Dexmedetomidine vs midazolam for sedation of critically ill patients: a randomized trial. JAMA 301, 489–99 10.1001/jama.2009.56 19188334

[24] Cole M.G. (2004) Delirium in elderly patients. Am. J. Geriatr. Psychiatry 12, 7–21 10.1097/00019442-200401000-00002 14729554

[25] Glass P.S. and Reves J.G. (1995) Drug delivery system to improve the perioperative administration of intravenous drugs: computer assisted continuous infusion (CACI). Anesth. Analg. 81, 665–7 7573990 10.1097/00000539-199510000-00001

[26] Sommer C. and Kress M. (2004) Recent findings on how proinflammatory cytokines cause pain: peripheral mechanisms in inflammatory and neuropathic hyperalgesia. Neurosci. Lett. 361, 184–7 10.1016/j.neulet.2003.12.007 15135924

[27] Grosu I. and Lavand'homme P. (2015) Continuous regional anesthesia and inflammation: a new target. Minerva Anestesiol. 81, 1001–9 25317576

[28] Tang C., Li J., Tai W.L., Yao W., Zhao B., Hong J. et al. (2017) Sex differences in complex regional pain syndrome type I (CRPS-I) in mice. J Pain Res. 10, 1811–9 10.2147/JPR.S139365 28831269 PMC5548282

[29] Gottschalk A. and Ochroch E.A. (2003) Preemptive analgesia: what do we do now? Anesthesiology 98, 280–1, author reply 1 10.1097/00000542-200301000-00047 12503013

[30] Pipolo C., Bussone G., Leone M., Lozza P. and Felisati G. (2010) Sphenopalatine endoscopic ganglion block in cluster headache: a reevaluation of the procedure after 5 years. Neurol. Sci.: Off. J. Italian Neurol. Soc. Italian Soc. Clin. Neurophysiol. 31, S197–S179 10.1007/s10072-010-0325-2 20464621

[31] Gearhart S.L., Do E.M., Owodunni O., Gabre-Kidan A.A. and Magnuson T. (2020) Loss of Independence in Older Patients after Operation for Colorectal Cancer. J. Am. Coll. Surg. 230, 573–82 10.1016/j.jamcollsurg.2019.12.021 32220448

[32] Cropsey C., Kennedy J., Han J. and Pandharipande P. (2015) Cognitive Dysfunction, Delirium, and Stroke in Cardiac Surgery Patients. Semin. Cardiothorac. Vasc. Anesth. 19, 309–17 10.1177/1089253215570062 26660055

[33] Whalin M.K., Kreuzer M., Halenda K.M. and Garcia P.S. (2015) Missed Opportunities for Intervention in a Patient With Prolonged Postoperative Delirium. Clin. Ther. 37, 2706–10 10.1016/j.clinthera.2015.09.012 26492795

[34] Tang C.L., Li J., Zhang Z.T., Zhao B., Wang S.D., Zhang H.M. et al. (2018) Neuroprotective effect of bispectral index-guided fast-track anesthesia using sevoflurane combined with dexmedetomidine for intracranial aneurysm embolization. Neural Regen. Res. 13, 280–8 29557378 10.4103/1673-5374.226399 PMC5879900

[35] Inouye S.K., Westendorp R.G. and Saczynski J.S. (2014) Delirium in elderly people. Lancet 383, 911–22 10.1016/S0140-6736(13)60688-1 23992774 PMC4120864

[36] Vasilevskis E.E., Han J.H., Hughes C.G. and Ely E.W. (2012) Epidemiology and risk factors for delirium across hospital settings. Best Pract. Res. Clin. Anaesthesiol. 26, 277–87 10.1016/j.bpa.2012.07.003 23040281 PMC3580997

[37] Su X., Meng Z.T., Wu X.H., Cui F., Li H.L., Wang D.X. et al. (2016) Dexmedetomidine for prevention of delirium in elderly patients after non-cardiac surgery: a randomised, double-blind, placebo-controlled trial. Lancet 388, 1893–1902 10.1016/S0140-6736(16)30580-3 27542303

[38] Reade M.C. and Finfer S. (2014) Sedation and delirium in the intensive care unit. N. Engl. J. Med. 370, 444–54 10.1056/NEJMra1208705 24476433

[39] Tasker R.C. and Menon D.K. (2016) Critical Care and the Brain. JAMA 315, 749–50 10.1001/jama.2016.0701 26903329

[40] Sobbi S.C. and van den Boogaard M. (2014) Inflammation biomarkers and delirium in critically ill patients: new insights? Crit. Care 18, 153 10.1186/cc13930 25042374 PMC4075413

[41] Shi C.M., Wang D.X., Chen K.S. and Gu X.E. (2010) Incidence and risk factors of delirium in critically ill patients after non-cardiac surgery. Chin. Med. J. (Engl.) 123, 993–9 20497703

[42] Nelson L.E., Lu J., Guo T., Saper C.B., Franks N.P. and Maze M. (2003) The alpha2-adrenoceptor agonist dexmedetomidine converges on an endogenous sleep-promoting pathway to exert its sedative effects. Anesthesiology 98, 428–36 10.1097/00000542-200302000-00024 12552203

[43] Mei X., Chen Y., Zheng H., Shi Z., Marcantonio E.R., Xie Z. et al. (2019) The Reliability and Validity of the Chinese Version of Confusion Assessment Method Based Scoring System for Delirium Severity (CAM-S). J. Alzheimers Dis. 69, 709–16 10.3233/JAD-181288 31127777 PMC7844342

[44] Shi S.M., Sung M., Afilalo J., Lipsitz L.A., Kim C.A., Popma J.J. et al. (2019) Delirium Incidence and Functional Outcomes After Transcatheter and Surgical Aortic Valve Replacement. J. Am. Geriatr. Soc. 67, 1393–401 10.1111/jgs.15867 30882905 PMC6612597

[45] Kimchi E.Y., Neelagiri A., Whitt W., Sagi A.R., Ryan S.L., Gadbois G. et al. (2019) Clinical EEG slowing correlates with delirium severity and predicts poor clinical outcomes. Neurology 93, e1260–e1271 10.1212/WNL.0000000000008164 31467255 PMC7011865

